# Mobilizing the banking sector for universal health coverage: a new frontier for public–private partnerships

**DOI:** 10.3389/frhs.2026.1750156

**Published:** 2026-04-02

**Authors:** Chidera Gabriel Obi, Faith Udochukwu Uzor

**Affiliations:** 1 Department of Parasitology and Entomology, Nnamdi Azikiwe University, Awka, Nigeria; 2 Access Bank Plc, Lagos, Nigeria; 3 Youth in Research Hub, Enugu, Nigeria; 4 Department of Business Education, Nnamdi Azikiwe University, Awka, Nigeria

**Keywords:** commercial banks, financial innovation, health financing, low- and middle income countries (LMICs), public–private partnerships, universal health coverage (UHC)

## Abstract

**Background:**

The attainment of Universal Health Coverage (UHC) remains difficult in most low- and middle-income countries (LMICs) due to gaps in health funding, high out-of-pocket spending and further worsening due to recent donor cuts. Existing literature predominantly focuses on traditional sources which include government budgets, donor aid, social health insurance, and household payments while the role of commercial banks as strategic health system financiers remains largely untapped beyond Corporate Social Responsibility (CSR) activities.

**Aim:**

This perspective examines how commercial banks can provide innovative solutions and utilize their untapped resources to become strategic partners in health financing which can be harnessed towards attaining UHC.

**Approach:**

Anchored in Financial Intermediation Theory and the World Health Organization's health financing framework, the paper reviews evidence from peer-reviewed literature, policy documents, and illustrative country experiences.

**Key arguments:**

Commercial banks possess significant liquidity, risk-assessment capacity, and extensive networks that can be potentially leveraged through health-targeted savings and insurance products, ESG-aligned health bonds, de-risked lending to health SMEs. Structured public–private partnerships can further improve health outcomes while maintaining profitability. Empirical examples from Nigeria and other LMICs demonstrate the feasibility of these approaches.

**Concerns:**

The involvement of commercial banks in health financing involves the risk of equity concerns (urban bias, over-indebtedness, technicality of the products and profit-equity alignment), especially in weak regulatory context while PPPs can carry political undertones with higher political risks.

**Recommendations:**

Commercial Banks and policy makers should promote health focused and inclusive products with literacy support, mobilize capital through bonds/guarantees, expand health SME credit, leverage PPPs, and monitor outcomes to mitigate risk.

**Conclusions:**

The integration of commercial banks into UHC as strategic partners can bridge financing gaps, improve health access, and strengthen health system resilience in LMICs, provided supportive regulation, ethical frameworks, and contextual adaptation guide their engagement.

## 1 Introduction

Universal health coverage (UHC) entails ensuring that the populace has access to needed health services without financial hardship through various programs and policies (1). It remains one of the core targets set in 2015 when the 2030 Sustainable Development Goals (SDGs) were adopted by all countries (2). However, across low- and middle-income countries (LMICs), there is a shortage of health financing, which continues to limit the ability to achieve UHC. In Africa, for example, most countries have failed to meet the Abuja Declaration target of allocating at least 15% of government expenditures to the health sector (3). This leads to financial constraints due to excessive out-of-pocket expenses. In Nigeria, for instance, 70% of medical costs are borne through out-of-pocket expenses, impoverishing the populace (4).

Conventional sources of health financing, which include taxation, social health insurance, private health insurance, and out-of-pocket payments (5, 6), have been essential but inadequate for attaining UHC. Limitations such as fiscal capacity, administrative inefficiencies, low enrolment rates and heavy reliance on external aid limit these conventional sources, leading to poor health outcomes and coverage (5, 6). Despite these challenges in conventional health financing, financial sectors such as commercial banks are not adequately utilized to address universal health coverage (7). Given the high liquidity and constantly improving risk assessment capacity of commercial banks, there is considerable potential for capital mobilization to strengthen the health system through diverse financial instruments.

There is a recent global trend, including in Nigeria, towards sustainable and environmentally friendly financing. This shift presents a unique opportunity for commercial banks to actively contribute to the health ecosystem. Through various health-focused products, such as healthcare infrastructure financing, microhealth savings schemes, and other ESG-aligned health portfolios, the banking sector can position itself as a strategic actor in achieving UHC. Despite these promising opportunities, there remain concerns regarding the alignment of profit-driven financial institutions with equity principles, highlighting the purpose of UHC, especially in areas with weak regulatory and financial protection.

Existing literature on health financing across low- and middle-income countries (LMICs) has focused mainly on taxation, donor funding, insurance schemes, and household out-of-pocket expenditure as major source of financing for health systems. However, commercial banks as a major actor for health financing has been limited beyond its role in corporate social responsibilities. This limits commercial banks as health system financiers rather than partners for UHC. The current dynamics presents the need for change towards this policy given the cut across funding sources which may impact the health systems. This raises the question: How can commercial banks in LMICs become fully integrated into national health financing strategies to advance Universal Health Coverage through innovative financial instruments and various risk sharing models? This prompts this perspective to examine how commercial banks in LMICs, particularly in Nigeria, can be mobilized as strategic partners in financing UHC through innovative financial instruments, risk-sharing models, and ESG-aligned investments.

## 2 Conceptual framework and theoretical anchoring

This Perspective is anchored in Financial Intermediation Theory (FIT), which conceptualizes banks (and other financial intermediaries) as institutions that mobilize surplus funds from savers and allocate the funds to borrowers or sectors with deficit (8). FIT identifies several core mechanisms through which banks and intermediaries create value, including the reduction of information asymmetries, risk pooling, transaction cost reduction, liquidity transformation (9, 10). Banks reduce information asymmetry through enhanced credit assessment, screening and monitoring, thereby reducing moral hazard and adverse selection (11), and manage risk by spreading the risk across portfolios (12). Banks also reduce transaction costs through scale and digital infrastructure. They perform liquidity transformation by channelling excess funds to borrowers (13, 14).

This theoretical framework can be extended to the health systems with similar market imperfections, suggesting that banks can play an integral role in improving health financing options. Banks can reduce existing information asymmetry associated with financing health-sector small and medium enterprises (SMEs) through credit assessments and monitoring (15, 16). They can also leverage risk pooling to offer insurance-linked products and structured financing that mitigate risk (7, 17). Furthermore, reducing transaction costs through savings mobilization and micro contributions can enhance payment arrangements. Banks can also enable liquidity transformation by directing excess funds to critical health services (1, 7). This anchoring connects directly with the core functions of health financing necessary to achieve Universal Health Coverage (UHC), including mobilizing funds, pooling of funds (risk pooling), and strategic purchasing as outlined in the WHO health systems framework (18). This perspective positions commercial banks as partners rather than peripheral financiers. It highlights how Banks can utilise the FIT framework to provide the structure for resource allocation in the health system while the WHO Framework guides how and where the intermediation can contribute to UHC.

The expansion of this framework is anchored through studies that have shown that enhanced financial development leads to improved health outcomes. For example, findings from forty-five countries from Sub-Saharan Africa by Musah et al. (17) have shown that improved financial development enhances health outcomes, especially in contexts where high out-of-pocket expenses, common in Sub-Saharan Africa (SSA), worsen financial outcomes. The study further stated that strengthening the financial system and public health financing would improve life expectancy and reduce mortality rates. The success of this framework can be seen in Barclays led $58.8 million bond issue with $48.5 million of which was designated as a social bond. Thihelped HealthRight in its fight against addiction, purchase of health equipment and its scalability through reduction in its interest expense. The oversubscription of the bond highlights promises for the sustainability of this framework in SSA (19, 20).

## 3 Analytical approach

This study adopts a structured narrative approach that draws on syntheses of peer-reviewed articles and relevant documents to examine the role of commercial banks in health financing. The article is guided by the Financial Intermediation Theory and the WHO health financing framework as baseline to outline health financing challenges in LMICs and how Commercial Banks can support the UHC through the core health financing functions.

## 4 Current landscape of health financing in LMICs

Funding from the government remains the major source of financing for most countries worldwide; however, increasing funding from the government alone will not be sufficient to cater to health financing needs or lead to UHC across Africa (21). These challenges require the need for more funding opportunities to help achieve UHC across Africa (22, 23). Despite the variety of health insurance schemes available, these schemes predominantly serve the formal sector (24). For example, in Nigeria, formal sectors such as banks, insurance companies, government workers, and top accounting firms have insurance coverage, whereas those in less formal sectors and SMEs generally do not have insurance coverage. This has led to high out-of-pocket spending as a source of health financing, resulting in a loss of income and poverty for the populace (25).

Across Africa, financing traditionally depends on four pillars: government budgets, donor assistance, insurance schemes, and household payments (24, 26, 27). These financing methods have helped bridge the gap in equitable health distributions. The government and donors are largely responsible for financing healthcare programs, health infrastructure and essential services (28, 29). However, health insurance schemes are utilized mainly by the formal sector and serve to pool risk and reduce direct financial burden (30), whereas out-of-pocket spending serves as the source of payment when other methods are limited and often leads to impoverishing the populace (31). Collectively, these mechanisms have formed the core structure for financing across various African countries.

In South Asia, there have been efforts to strengthen the health system through social health insurance (SHI). For example, In Nepal, recent evidence from the SHI suggests an expanded population coverage and service utilization, though persistent challenges in enrollment, sustainability, and benefit adequacy remain (32, 33). Similarly, in Indonesia, the mandatory National Health Insurance Scheme which was initiated in 2014 has helped improve health care utilization among women (34). These further reflect broader LMIC trends, progress in coverage however gaps persist in financial protection and sustainability due to constrained domestic resources and reduced external aid (35).

Despite the progress made through traditional health financing mechanisms, several limitations continue to hinder the attainment of UHC across Nigeria and other African countries. Government spending and donor funding can often be unreliable due to economic and political environments. For example, in the past two years, there have been shortages in donor funding due to withdrawals of some funding initiatives across the LIMCs. These cuts have led to disruption of the fight against malaria, HIV and tuberculosis, among other health implications (36). Insurance coverage tends to be limited, as it does not cover chronic diseases, whereas out-of-pocket expenses tend to affect the economic levels of households. These limitations and inefficiencies highlight the need for innovative financing methods to improve health outcomes. This provides an opportunity to influence banking sector liquidity to bridge existing gaps. These can be through providing working capital for SMEs and financing healthcare infrastructure for the health sector, partnering health insurance companies to provide credit and insurance options for the informal health sector.

## 5 Opportunities for the banking sector in advancing UHC

The various mechanisms highlight the huge potential of the banking sector in health financing; however, without appropriate safeguards, these financial solutions may further create inequalities in healthcare services.

### 5.1 Health-targeted savings and micro health products

The application of FIT highlights to health-targeted savings products highlights how small, consistent deposits can lead to increased liquidity and reduction of cost while facilitating risk pooling. Through combination of small deposits towards pooled insurance thereby allowing Banks to limit individual exposure to catastrophic spending. These health-targeted savings and micro health products are designed to help households plan and save for the purpose of healthcare expenses.

The evidence suggests that these products can enhance both financial protection and health access. A systematic review highlighted that microfinance interventions, such as savings-based schemes, can improve households’ capacity to manage financial shocks and health emergencies (37) and reduce out-of-pocket spending and devastating health expenditures (38). These findings indicate that health-focused micro savings can enhance financial inclusion.

This presents a strategic opportunity for Banks to integrate health-targeted micro savings products within existing retail banking systems. By collaborating with insurance providers and leveraging digital banking platforms, such products can link savings directly to health insurance, creating a sustainable structure for expanding healthcare financing. For example, Rwanda's community-based *Mutuelles de Santé,* which combines micro savings with subsidized insurance, achieved 91% population coverage and reduced health-related impoverishment by 70% (39). Although not bank-led, this initiative illustrates how structured prepayment and pooled financing mechanisms can expand coverage and reduce financial hardship. Thus, in the context of the ongoing financial inclusion drive across Nigeria and Africa, integrating health-targeted savings and micro health products into banking systems will enable households to better prepare for unexpected medical costs such as accidents, emergency surgeries, childbirth complications, and hospitalizations.

### 5.2 Health bond and environmental, social, and governance (ESG) aligned investments

From a FIT standpoint, ESG-aligned investments and health bonds operationalise risk pooling and liquidity transformation by mobilising long-term resources for the health sector. Banks can actively promote ESG-aligned investments by influencing corporate practices and investment flows into social and environmental determinants of the health of their customers through various lending guidelines (40). This can help improve health outcomes and enhance the sustainability of the financial system itself. Through sustainable finance frameworks, various ESG factors are increasingly integrated into financial strategies that support sustainable development while addressing environmental, climate, and social challenges. These include a range of financial products and services such as green bonds, impact investing, and carbon financing and trading (41, 42). The relevance of this framework is illustrated by a $53 million social bond issued by Barclays for HealthRight 360, which provided long-term funding for community health facilities in California (19). This highlights how these bonds can lower borrowing costs for essential healthcare providers.

Adherence to these ESGs can also be beneficial to banks through access to capital, long-term value creation, reputation and brand enhancement and risk management (41, 42). Within the FIT framework, the introduction of health bonds enables liquidity transformation by converting short-term liabilities into long-term healthcare infrastructure financing These bonds can address healthcare gaps by supporting investments in primary healthcare infrastructure, closing critical primary healthcare gaps and financing digital health expansion and/or health SMEs. Through investments in health bonds, investors can support initiatives that directly benefit public health and contribute to sustainable economic growth and resilience.

The health bonds illustrate liquidity transformation in the FIT framework by converting short-term deposits into long-term health infrastructure financing, while risk pooling is achieved through diversified bond portfolios that distribute repayment exposure across investors.

### 5.3 Financial literacy for health resilience

Studies have shown that increasing financial literacy can have profound effects on individuals’ physical and mental health by promoting better financial decision-making and stress management (43, 44). However, financial literacy for health resilience goes beyond having access to financial institutions and utilizing the various savings and credit facilities available. It also encompasses various health-related financial plans, such as preventive health spending, insurance awareness and preparedness for emergencies, which are essential components of household financial resilience (45). These financial plans help households avoid excessive out of pocket spending while ensuring adequate health coverage thereby supporting UHC (46). From a FIT perspective, financial literacy reduces information asymmetries between households and financial institutions, thereby enhancing the efficient allocation of financial resources toward health-related expenditures (47). The understanding of various financial products and services enables households to participate in risk pooling mechanisms and utilise formal financial services effectively.

In Nigeria, the Central Bank of Nigeria (CBN), through the National Financial Inclusion Strategy (NFIS) 2024, drives health resilience by targeting 40% formal insurance and pension coverage, 60% savings usage, and 70% digital payments to enable affordable health protection, emergency buffers, and seamless access to care (48). These targets recognize financial literacy as a public health tool. Within this framework, banks serve as critical intermediaries in embedding health-oriented financial education into customer engagement strategies, product design, and SME support programs. Through collaboration with international donors, NGOs and other public health institutions, the banking system can utilize financial literacy to improve health resilience including channelling the surplus funds towards health aligned deficits in support of UHC highlighting the bank liquidity transformation role in UHC.

## 6 Case perspective: Nigeria

In Nigeria, commercial banks act as active private sector contributors to social welfare and improve health outcomes beyond their core financial services. These institutions have increasingly participated in social initiatives that indirectly support public health outcomes (49). The top five Nigerian banks, popularly known as FUGAZ (First Bank, UBA, Guaranty Trust Bank, Access Bank, and Zenith Bank), are at the forefront of health-related corporate social responsibility (CSR) initiatives. These banks have carried out various health-related projects aimed at improving children's and women's health, free testing, consultations, and community engagement (50–52). These initiatives have indirectly contributed to solving public health problems by promoting preventive care, community sensitization, and support for vulnerable populations. These CSR initiatives can be further streamlined into PPP initiative mirroring the success of the CACOVID to form a long term and sustainable partnership ensuring improved health outcomes such as seen in National Referral Hospital Network while learning from challenges encountered from the program (53).

In addition to these health-related CSR projects, the Nigerian banking sector also contributes to public health improvement through various empowerment schemes and financial inclusion programs. These financial solutions aid in building resilience among communities and households, enabling them to afford various necessities to improve their living. For example, the Access Bank has a W Initiative, which provides credit facilities to women for fertility, child delivery, paediatric care, cancer treatment and other specialized procedures and provides women entrepreneurs with tailored credit facilities for their businesses (54). These enable women including women owned businesses to access credit, which ultimately increases their available funds for healthcare and health treatments at lower rates, increasing their access to health coverage. Similarly, the Guaranty Trust Bank and Zenith Bank have supported SMEs through dedicated financing platforms that include community pharmacies, diagnostic centers, and healthcare startups. By strengthening the financial capacity of these businesses, banks contribute to sustaining the healthcare ecosystem and expanding access to essential medical services, particularly in underserved areas. These efforts align with the Central Bank of Nigeria's (CBN) financial inclusion strategy, which emphasizes inclusive economic participation as a pathway to improve social and health well-being. These strategies are in line with successful models across Africa such as the as Rwanda's Mutuelles de Santé, where community linked savings and insurance reduced impoverishment from health costs (39). Products which encourage community linked savings and insurance can help reduce catastrophic out of pocket spending especially among the informal sector in Nigeria.

Over the years, streamlined health financing schemes aimed at strengthening private public partnerships to improve health systems have been developed. For example, the CBN earmarked 100 billion naira to be disbursed to eligible healthcare institutions toward improving health systems through financial institutions at specialized rates in response to the COVID-19 outbreak (55, 56). These low interest funds enabled public and private health sector players to access funds, which in turn improved health infrastructure across the country. However, given that new applications for this initiative have been halted by the Central Bank (57), there is a need for commercial banks to provide specialized loans mirroring similar low interests to sustain progress in health system development, potentially in collaboration with donors and health organizations.

## 7 Challenges and risk considerations

Globally, there are limited regulations mandating or incentivizing commercial banks to lend to the health sector. Banks face various regulatory and structural barriers, such as limited credit risk data, challenges in assessing repayment capacity due to poor financial records, inadequate investment instruments, weak legal frameworks, and poor risk-sharing mechanisms, which make health investments appear high risk and unattractive to financial institutions (7, 58). Consequently, these challenges not only restrict the flow of private funds into health but also may prevent viable health institutions from accessing the quality financing needed to expand infrastructure and sustain operations.

Health project financing also presents reputational and portfolio risks for financial institutions. These risks stem from limited collateral, unpredictable cash flows, long breakeven cycles, and the sensitivity associated with health services, given that health services deal directly with human life. Additionally, the specialized nature and uncertain resale value of many health infrastructure assets can limit collateral realization, thereby increasing risk exposure for financial institutions (15, 17). Consequently, banks may face reputational scrutiny from the host community if a financed health institution fails, even when all proper due diligence has been conducted. Despite their vulnerability to shocks, SMEs have fewer resources and weaker resilience than larger firms do (59), which heightens credit risk for many health businesses, especially since the majority of health enterprises fall within the SME category (60). Even large-scale health PPPs are vulnerable to these shocks as seen in Lesotho where the inability of the PPP contract to adapt to significant public-sector wage increases led to a massive loss of skilled personnel to the government, illustrating how contractual inflexibility can heighten long-term credit and operational risk (61). This elevated risk environment can discourage banks from increasing their exposure to the sector, particularly in the absence of mechanisms to mitigate potential losses.

To encourage banks to lend to enterprises for public health infrastructure and working capital while mitigating the risks that may arise from such lending, there is a need for effective risk-sharing mechanisms. These include risk-sharing facilities, guarantee funds, donor-supported instruments, and government-backed bonds (62–64) targeted at the health sector. An example is initiative by the Medical Credit Fund in East Africa, which uses donor-supported guarantees to reduce the perceived risk for local banks share risk thereby improving lending especially to health institutions with inadequate funding (64). Although these schemes do not replace routine credit checks or due diligence, they provide additional comfort by cushioning potential losses and reducing perceived credit risk. This added assurance can stimulate increased lending to the sector, ultimately supporting improved health service delivery and coverage.

## 8 Scalability and contextual considerations

Sustained commitment from government and political office holders to healthcare financing is essential for the long-term sustainability of health interventions. Such commitment helps ensure that interventions including government-backed guarantees and healthcare subsidies are maintained across successive election cycles thereby reducing policy uncertainty. In the absence of consistent government support to commercial banks and other health financing institutions through incentives such as tax credits linked to healthcare financing thresholds, there is an increased risk of stalled or failed interventions. McDaid and Park suggest that fiscal incentives are important in stimulating sustained intersectoral engagement (65) and fostering dialogue between finance and health authorities can help align budgets and fiscal space for health (66).

Beyond political involvement, supportive regulations and economic conditions towards these financing initiatives are integral to scaling these financing initiatives. Inflation and exchange rate fluctuation is common in LMICs as evidenced by studies in Nigeria (67) and can undermine financing initiatives unless mitigated through regulatory frameworks and bank-level risk management, including de-risking instruments like currency hedging. Furthermore, fixed monthly insurance contributions may be unsuitable for populations with irregular incomes. Studies in Indonesia and Kenya highlighted the challenges of these fixed monthly payments among the informal sector workers, pinpointing the need for more flexible options (68, 69). Finally, the digitalization that has sprung up across LMICs highlights the need for these interventions to be aligned with digital infrastructures to ensure wider reach and lowering cost.

## 9 Ethical considerations: equity, access, and financial protection

The involvement of commercial banks in health financing highlights important ethical questions related to equity, access and financial protection. Greater involvement of commercial banks in health financing may introduce several structural risks for national health systems, particularly in LMICs where regulatory capacity may be limited (70). One potential concern is the inequitable distribution of financing opportunities, as banks may prioritize urban areas with better repayment capacity and financial infrastructure (71, 72). Studies indicate that private capital tends to favor urban and high-income areas (71, 72) which might defeat the equity and access objectives of UHC.

At the household level, there are also potential financial risks associated with banking sector participation if proper mitigations are not in place (73). Over-indebtedness is one of the major risks associated with this type of funding particularly for vulnerable households, if repayment obligations exceed household income capacity, especially when borrowing occurs across various financial institutions (74). In addition, high interest rates associated with such loans may worsen financial stress on households rather than improve healthcare outcomes. There also exists possibility of exclusion clauses in the health insurance that limit coverage for preexisting conditions, which ultimately limits access to care and the broader goals of UHC (75, 76).

Institutional and governance constraints may further affect the effectiveness of banking-sector engagement in health financing. In many LMICs, Ministries of Health and health boards may have limited capacity to monitor complex financial instruments thereby creating challenges in ensuring accountability and transparency (77). This highlights the need for stronger collaboration between the health and finance ministries in health financing activities. PPPs in health infrastructure may also have political undertones, where private partnerships might be politically motivated, leading to increased scrutiny and higher political risk (78). These dynamics highlight the need for strong regulatory coordination between financial regulators, ministries of health, and development partners to ensure that banking-sector participation aligns with national UHC objectives.

## 10 Recommendations for mobilising the banking sector to support universal health coverage (UHC) in low- and middle-income countries (LMICs), with a focus on Nigeria

1. Design and Promote Inclusive Health-Focused Financial Products with Integrated Financial-Literacy Support: FIT framework indicates that bundling micro savings account with health insurance options enhances risk pooling by spreading the healthcare financial risk across many households. The digitalization through digital micro savings platforms may help improve efficiency. Leveraging the large customer base of banks and the drive towards digitalizing the financial sector, there is need to partner with health insurance companies to bundle low-cost micro-savings accounts with basic health-insurance coverage options. These products target semi-skilled individuals and those in the informal sector. Evidence from Rwanda shows that micro-saving integrated with health insurance improved health outcomes by improving access to healthcare and reducing outrageous out of office spending (39). Similar models can leverage the growing financial digital platforms to form collaborations between commercial banks and insurance companies to improve access to health care. Targeting Micro Health Insurance at low-income households and specific cultural locations has been shown to prevent disastrous out-of-pocket spending (63). By exploiting the digital banking platforms to enable automatic premium deductions and real-time tracking of health-savings options and available health facilities, the individuals are closer to having proper health coverages. These options can also be piloted towards high-need regions and scale successful models through the Central Bank's financial-inclusion initiatives. To maximise uptake and sustained use, banks should simultaneously integrate health-resilience financial-literacy modules into their customer education and onboarding programmes. Through collaboration with public-health agencies and NGOs, simple and culturally relevant content on preventive care budgeting, insurance literacy, and emergency-fund planning can be developed. Participation and comprehension can be tracked with options for feedback to help to demonstrate impact and refine programme design.
2. Mobilise Private Capital through Health-Focused Bonds, De-Risked Lending, and Credit Guarantees: Health Bonds and ESG-aligned instruments can lead to liquidity transformation through mobilization of long-term capital for health infrastructure and services (7, 58). Banks can create Health Impact Bonds (or broader ESG-aligned instruments) that earmark proceeds for primary care infrastructure, digital health platforms, and health SME financing. These bonds should align bond issuance with national ESG frameworks to attract socially responsible investors and multilateral funding and protect the environment. Access Bank's successful issuance of the first Green bond in Africa along with the strong investor demand it attracted (79) which has been followed by other bank green bond issuance across Africa highlights the potential of such instrument to finance eco-friendly health projects while still delivering competitive returns for banks. To encourage banks to increase lending and reduce the inherent risk that is associated with financing health institutions, these bond issuances should be paired with robust risk-sharing mechanisms including partial credit guarantees, donor-backed first-loss facilities, and government co-financing. When these instruments are paired with donor-backed guarantees, it can provide private capital for health infrastructural development (7). There is need for the government in collaboration with the Central Bank and other players to establish and expand guarantee funds or partial credit guarantees which can help to mitigate default risk for health sector loans. In addition to these guarantees, banks should also develop standardized credit risk assessment tools tailored to health SMEs, drawing on data from existing micro-finance and health insurance schemes. Furthermore, multilateral donors should be encouraged to provide first loss coverage for health infrastructure projects, reducing perceived risk for commercial lenders and making the sector more attractive for private capital (64, 80). The FIT framework highlights how Banks can utilize these instruments to mobilize private capital for health financing through risk pooling, liquidity transformation and improved information screening thereby leading to UHC.
3. Expand Credit Facilities for Health-Related SMEs and Women-Led Enterprises: Expansion of Credit Facilities to health-related SMEs enables liquidity transformation whereby commercial banks transfer excess deposits into financing for healthcare systems. Through utilisation of the various Government backed health bonds and guarantees, the Banks should design low-interest, collateral-flexible loan products for community pharmacies, diagnostic centres, maternal-health clinics and other health clinics for healthcare infrastructure and working capital need. These facilities should also extend to offer preferential rates for women-owned health businesses, linking loan approval to business-development support and mentorship. These support and mentorship will enable banks to better assess borrowers to reduce information asymmetry.
4. Leverage Existing Public-Private Partnership (PPP) Frameworks: Sustainable PPP frameworks can strengthen risk pooling and improve liquidity by enabling banks to mobilize private capital for health infrastructure while distributing financial risk among multiple stakeholders. In Nigeria, PPP frameworks played a significant role during the COVID-19 response, with banks through initiatives such as CACOVID, working within PPP structures to strengthen health outcomes (53). Building on these precedents, the bank can align its health financing with various health-sector PPP policies, targeting projects such as hospital construction, equipment leasing, and tele-medicine networks. Enhanced coordination between the Ministry of Health, and private-sector actors should be promoted to streamline approval processes for health-sector loans to improve health outcomes. In addition, the co-payment structure can be integrated into the PPP arrangement to balance sustainability with equity. This structure can help the commercial banks to share the inherent financial risk while reducing out of pocket expenditure. This can build on the existing NHIS/NHIA, where a 10% co-payment on drugs is already applied for insured beneficiaries to promote individual commitment and affordability (81) and further extend beyond salaried workers
5. Monitor, Evaluate, and Report on Health-Financing Outcomes: Banks can provide adequate and sustainable frameworks to ensure proper monitoring and reporting on health sector and health financed projects to ensure adequate and transparent data. This is in line with regulations which ensure that banks collect data and report various financial transactions to regulatory authorities (82). This becomes more important as the success of health financing initiatives depends on continuous monitoring to identify challenges and measure outcomes (83). Banks should establish a standardized set of health financing metrics, developed in collaboration with relevant health institution such as the increase in health-insurance enrolment, number of health facilities financed and increase in workforce to assess the impact of banking interventions. These can be enhanced by publishing annual segments on health projects financed to enhance transparency, attract further investment, and inform policy adjustments which helps reduce information asymmetry. Through these findings, the Government can incentive structures for continuous improvement while the Banks can refine product design and risk models.

These recommendations can help transform commercial banks from passive financial intermediaries into active partners in the quest for Universal Health Coverage. By leveraging their core financial intermediation functions which includes liquidity transformation, risk pooling, information screening, and transaction cost reduction, the banks can mobilize funds while improving financial access in health financing thereby closing the financing gap in LMIC health systems.

## 11 Conclusion

The attainment of UHC across LMIC requires a cross-sectional collaboration that extends far beyond Governments, Ministry of health and traditional donor. Recent donor cuts and funding shortages from the Government have exposed the limits of conventional financing models while it presents an opportunity for banks. However, while these opportunities exist, they should be viewed as complementary mechanism within public health financing rather than substitutes. Their vast liquidity, networks, increasing drive towards ESG and risk assessment capabilities equips the banks to bridge existing financing gaps in the health system through various innovative such as insurance bundles, health driven bonds and increased lending to health SMES. While structural, regulatory, and market barriers exist, these can be mitigated through policy interventions, risk-sharing instruments, and alignment with public health objectives.

## Data Availability

The original contributions presented in the study are included in the article/Supplementary Material, further inquiries can be directed to the corresponding authors.
